# Personal observations on COVID-19 and the conduct and application of biomedical science

**DOI:** 10.1098/rsfs.2021.0053

**Published:** 2021-10-12

**Authors:** J. C. Smith, David W. Goodhew

**Affiliations:** ^1^ Developmental Biology Laboratory, The Francis Crick Institute, London NW1 1AT, UK; ^2^ Wellcome Trust, 215 Euston Road, London NW1 2BE, UK; ^3^ Latymer Upper School, King Street, London W6 9LR, UK

**Keywords:** COVID-19, conduct, applications, biomedical, science

## Abstract

We begin by describing our observations of the ways in which the conduct of research has changed during the COVID-19 pandemic and go on to comment on the quality of the scientific advice that is provided to UK citizens, and especially to schools. Researchers, like many, have suffered from the effects of the pandemic. Those hardships notwithstanding, we suggest that research into COVID-19 has benefitted from a ‘seed corn’ of discovery science that has provided the basis for routine diagnostic PCR and antibody tests; for structural analyses of the way in which the SARS-CoV-2 virus interacts with cells; for the development of new treatments (and the debunking of ineffective ones); for studies of the genetics of susceptibility to SARS-CoV-2; and for the development of vaccines. The speed of dissemination of research has benefitted from the widespread use of pre-prints, and researchers and funders have become more nimble in their approaches to research and more willing to change their priorities in the face of the pandemic. In our experience, the advice provided to schools on the basis of this research was, however, often published at the last minute and was frequently flawed or inconsistent. This has led to a widening of the attainment gap between children from disadvantaged backgrounds and their peers and it has exacerbated the digital divide and holiday hunger. The consequences will be felt for many years to come and will jeopardize diversity in research and other careers.

## 1. Introduction

This paper contains the personal perspectives of the authors—one a researcher and funder of research (J.C.S.), and one an educator (D.W.G.). We focus in particular on changes in the way science has been carried out during the COVID-19 pandemic and on the scientific advice that has been provided to schools in the UK—and how the latter might have been improved. As in other areas, research and education have been hard-hit by the pandemic: mental health has suffered [1]; research has had to be put on hold; workloads have increased; and caring responsibilities have fallen particularly on women [2]. At the end of this paper, we highlight another consequence of the pandemic: the increased attainment gap between disadvantaged pupils and their peers, and the consequence this will have on diversity in areas including science and research.

## 2. Science: a good COVID?

A lot has been written about the way scientists have responded to the appearance of SARS-CoV-2, and (if anything positive can be said to have come from the pandemic) the consensus is emerging that the urgency and focus imposed by COVID-19 have in some respects changed the way we do research for the better. The first part of this paper discusses this shift in scientific culture, what it involved, and how it might be maintained and developed in the future.

This is not the place for a history of COVID-19, but it is worth recalling that the first cases of the disease in Wuhan were reported to the World Health Organization at the end of December 2019 [3]. Within 10 days, the causative agent was identified as a novel coronavirus, and the genome sequence was made publicly available on 10 January. The first case in the UK was announced on 31 January 2020, and at the beginning of April, there were over one million cases worldwide (including UK Prime Minister Johnson) and over 75 000 deaths [4].

Individual governments may have tarried, and in doing so wasted some weeks or even months, but the ability of SARS-CoV-2 to spread so quickly, its high infection fatality rate (when compared with influenza, if not with other coronaviruses like SARS and MERS [5,6]), and (bluntly) the fact that it has affected people in high-income countries as well as in low- and middle-income countries, elicited the most extraordinary shift in scientific priorities and the rate of scientific achievement. Within a year, following the identification and sequencing of the virus, routine diagnostic PCR [7] and antibody-based [8] tests were established; structural analysis of the SARS-CoV-2 spike protein revealed how it interacts with angiotensin-converting enzyme 2 (ACE2) [9]; treatments involving existing drugs were demonstrated to be effective (dexamethasone and remdesivir) [10,11] or not (hydroxychloroquine) [12]; new treatments were developed (such as antibody therapy) [13]; the genetics of susceptibility was analysed [14]; vaccines were developed [15]; extensive genome screening was carried out [16]; and variants were identified with different transmissibilities and different effects on different age groups [17]. And much of this work, around the world, was carried out in the background of extraordinary political shenanigans. Never was there a better time to heed Sydney Brenner's maxim—*do the best experiments you can and always tell the truth* [18].

These extraordinary achievements of 2020 did not depend on fundamental advances in scientific understanding. Rather, they depended on two things. The first was a long history of discovery science—science aimed solely at understanding how the world works. All the achievements listed above are built on existing knowledge. We knew how to sequence nucleic acids; we knew how to do structural biology; vaccine development had been worked on for many years, including the concept of RNA vaccines; and we knew how to do randomized controlled trials. In other words, most, if not all, of the know-how needed by the scientific community to respond to the pandemic was already there, in the scientific literature, and the task was to mobilize this knowledge, with great skill and at great speed, in pursuit of the prevention and treatment of COVID-19.

But this mobilization might have been the hard part. Coordination, collaboration, speed and nimbleness were of the essence, and science is not always coordinated, collaborative, speedy or nimble. Indeed, there are several features of the way science usually works that mitigate against these objectives.

One of these features is the scientific career structure. Individual career success, together with any fame and fortune it may bring, depends on being first to a discovery. This is one of the reasons (in addition to the intrinsic interest of the work, of course) that researchers work so hard, and often encourage their immediate colleagues to do the same. As long as it is not taken to extremes this is no bad thing, but the desire to be first does mean that the incentives to share results and reagents before publication are few—why give your competitors an advantage, and the opportunity to ‘scoop’ you? The more enlightened researchers (often those already in a position of some seniority) recognize the value of sharing and will encourage it in their mentees, but as Raff [19] has said, the instinct is not to give—you have to be taught to share. This desire to keep stuff to oneself mitigates against coordination and collaboration, and it is rare indeed that those working on the same problem exchange ideas. (Although things may have been better in the ‘old days’. When J.C.S. was struggling to purify a so-called mesoderm-inducing factor in the late 1980s [20], he was enormously grateful to colleagues and ‘competitors’ like Igor Dawid, Marc Kirschner and Doug Melton for their support and encouragement.)

Even formal collaborations can be fraught with difficulty. The most common way in which academic credit is assigned, at least in the biomedical sciences, is through one's position in the authorship list of the paper that might be published. As a measure of intellectual contribution, it is hard to imagine anything cruder and less nuanced, and disputes between authors can be bitter, protracted and unhelpful [21].

Thus, the scientific career structure hinders collaboration. A factor mitigating against speed is the publication process, which can be painfully slow. It can take weeks for editors to decide whether to send a paper out for review; it can take months for the reviews to be sent to the authors and it can then take many months to do the additional experiments the referees insist will improve the work and make it worthy of publication. It can take so many months, in fact, that authors may even suspect that a referee is trying to delay, by over-zealous reviewing, the publication of their rival's work. The consequence of all these steps is that a paper may take well over a year to be published. If this had happened to the first papers describing SARS-CoV-2, the work might only have just been appearing alongside this article.

Finally, to respond to COVID-19, it was necessary for researchers to be nimble in changing the direction of their research. Under normal conditions, the consequences of changing one's research topic are profound: such a change would result in a hiatus of some years while the new research was set up, and there would be another year of struggling to get that first paper published. In a career in which a steady stream of publications is a *sine qua non* of success, and in which one is only as good as one's last paper, the risk of shifting research direction is great, and can usually only be done by senior researchers through a gradual and judicious closure of the old work and the gradual starting of the new.

## 3. Things have changed—and the response of the Francis Crick Institute

The pandemic changed all this. Faced not with the vicissitudes of a scientific career but with a global pandemic in which almost 4 million have now died, the research community pulled together in an extraordinary fashion, recognizing the importance of collaboration and sharing, of the need for a change in the dissemination of research and of the need to change field.

One change was to do with sharing and the dissemination of research. The importance of rapid and open access to data had been emphasized particularly by John Sulston in the context of the sequencing of the human genome [22], and this principle transformed genome research. But the idea was not adopted throughout biomedicine, and in many areas of science there remained a proprietorial attitude towards one's data. This began to shift with the principle of open access, championed by the Wellcome Trust and now by cOAlition S [23], but an even more profound shift came with the rise of pre-prints. Pre-prints make it possible for authors to make their papers publicly available before they are peer-reviewed, allowing the research community to discuss the data immediately and even to offer advice to authors before they submit to a peer-reviewed journal. Pre-print servers take great pains to emphasize that the papers have not been peer-reviewed, but the availability of the results, and their ready accessibility to the research community, encourage open discussion and allow authors to improve their papers before submission. Although peer review remains important and is the ‘gold standard’ to which researchers and policy-makers adhere, pre-prints are of great value in providing speed and permitting expert public scrutiny [24–26].

And pre-prints came into their own during COVID-19. The opportunity to share results quickly, and to allow the entire research community to analyse the data, made a huge difference to the dissemination of SARS-CoV-2 research, as did the propagation of the results by social media such as Twitter [27]. It is quite possible that the rapid publication of COVID-19 research through pre-prints will have saved many lives.

The ability of researchers to redirect their research during the pandemic is well illustrated by the Francis Crick Institute, where outstanding discovery scientists quickly turned their hands to the new challenge of COVID-19. The story began with the recognition by the Crick's clinical researchers, in discussion with University College London and University College London Hospital, that a testing pipeline was needed for frontline NHS staff and for care homes and their patients. It was also important that Crick researchers themselves be tested, to ensure that the Crick was a safe place to work. Three hundred Crick employees contributed to this work, including those working in the Institute's science technology platforms, and in doing so, they supported 10 hospitals and 150 care homes. The Institute's scientific expertise allowed the careful evaluation of testing methods and the development of downloadable Standard Operating Procedures. Together, these established the Crick as an extension of the accredited laboratories at the Health Services Laboratory [28].

At the same time, much research at the Crick switched to the study of the SARS-CoV-2 virus, and not just in the laboratories whose expertise was already in infectious disease. Examples of such research included questions of how the immune system responds to the coronavirus [29,30], and why some cases are so much worse than others [31,32]; studies of how the virus interacts with cells, including structural analyses of the interaction between the virus spike protein and ACE2 [9]; work on the evolution of SARS-CoV-2 [33,34], on its transmissibility [35] and on its susceptibility to vaccines [36]; and the question of how COVID-19 affects those who are already ill, and especially cancer patients [37].

Bringing all this work together, the Crick has also embarked on its legacy study, to understand what makes people vulnerable to SARS-CoV-2. The Institute's testing partnership has so far accrued over 400 000 samples, from healthcare workers and its own staff. The Crick will now, with informed consent, match these samples with individuals to study how factors like age, sex, ethnicity and medical history affect the risk of infection. The Institute will take further samples over the next 2 years to provide information about emerging variants and their transmissibilities, symptoms and susceptibilities to vaccination [38].

The Crick's researchers have been able to carry out these studies because they work in a core-funded institute which has allowed and even encouraged its staff to contribute to our understanding of SARS-CoV-2 and COVID-19. In a similar fashion, science at the Wellcome Sanger Institute has also turned its attention to COVID-19 and is sequencing thousands of virus genomes to trace transmission, inform public health measures and monitor for new variants [39]. In principle, it is harder for university staff, funded on research grants, to change their direction of research, but both Wellcome and the Medical Research Council have offered extensions to grants and have considered requests to change research to activity aimed at understanding COVID-19 and to contribute to its treatment and prevention.

## 4. How does science affect people?

Science and scientists responded well to the challenges of COVID-19, but it proved more challenging, in a changing landscape, to turn research results into clear advice to the public. Schools and schoolchildren were particularly badly affected in this regard, partly because of the initial lack of data about the impact and transmission of COVID-19 among children, but also because the guidance provided by the UK Department for Education (DfE) did not command the trust and confidence of the teaching profession: as we discuss below, it was often impractical and published at the last minute; it frequently contained errors and required significant revision; and sometimes it was internally inconsistent or contradicted guidance from Public Health England or the scientific community. For head teachers deemed to be *in loco parentis* of the children in their care, the release by the DfE of more than 200 policy updates between 18 March and 18 June 2020 [40] provided additional and unnecessary stress.

Some of the mitigations recommended by the DfE, like hand washing and frequent surface cleaning, were straightforward to implement. However, others were impractical. Even reducing social distancing from 2 m to just 1 m, whether in playgrounds or especially in classrooms, would have required a doubling of the number of classrooms and staff and would have made little practical difference [41]. And although the year-group ‘bubbles' proposed by the DfE did allow schools to return in September 2020, preventing closure of the entire school should there be a year-group outbreak, transmission *per se* could not be prevented. It was clear early on that the clinical risk to children and teenagers from COVID-19 was (mercifully) very low, but less clear at the time was the role of children in transmission, and especially asymptomatic transmission. In fact, schools proved not to be ‘engines of transmission’ but merely to reflect local prevalence; children are likely to be asymptomatic and have a much-reduced risk of transmission [42].

The uncertainties facing schools were exacerbated by hearing independent advice from groups other than the DfE—this included the emerging international consensus and the UK's own ‘Independent SAGE’ [43], a group of scientists working together to provide independent advice to the UK government and public on how best to respond to COVID-19, thereby complementing the government's own Scientific Advisory Group for Emergencies [44]. In response, D.W.G. found it necessary to create his own risk assessments on evidence and recommendations from Harvard Medical School, the World Health Organization, the Royal Society's DELVE group and the *British Medical Journal*. The availability of the Public Health England and Office for National Statistics dashboards was particularly valuable and allowed him to make a well-informed and independent view about the viability of reopening schools in January 2021. The wearing of masks in the classroom provides a telling example. Amid much confusion [45], face masks were finally recommended for use in schools by the DfE in March 2021, but Latymer Upper School had mandated wearing face-coverings from September 2020.

Schools provide a powerful example of the importance of building the best scientific advice, and of tailoring that advice to circumstances. It is a sad irony that the group within society that is at least risk of serious illness from COVID-19, or of transmitting it, has been one of the most badly affected, not just educationally but economically. As Sir Peter Lampl, founder and chairman of the Sutton Trust and chairman of the Education Endowment Foundation, has said: ‘By the time schools reopen, children and young people will have faced almost a year of learning disruption. The repercussions of these months of lost learning are devastating and will be felt for a lifetime, especially by those from low-income backgrounds’. We agree that the consequences of the cancellation of exams and of the differential learning loss caused by remote teaching will be felt for many years to come. The pandemic has emphasized the digital divide and holiday hunger and widened the attainment gap between disadvantaged pupils and their peers. This is tragic on an individual level and will have the additional effect of reducing the diversity in scientific and other workforces that is so necessary for success [46,47].

## 5. Conclusion

What, from our perspective, have we learnt from the COVID-19 pandemic? From the point of view of research, we first note that it is investment in discovery science that has allowed researchers to make such dramatic progress in our understanding of SARS-CoV-2. Without this ‘seed corn’, as it has been termed by Nurse [48], there would be no applied research and there would have been no rapid response to COVID-19. And, equally, without discovery research, there may be no rapid responses to the challenges of the future, including not only infectious disease, but also two other challenges recently identified by the Wellcome Trust as strategic priorities—climate change and mental health [49]. We hope that COVID-19 will serve as a warning that science is the only way out of the health challenges of the future and that this warning, like so many warnings of the past, will not be ignored.

The second lesson concerns the way science is done. Freed from self-imposed constraints to do with career advancement and with the publication process, and with the new-found ability to change research direction, research became more collaborative, more speedy and more nimble. These three qualities allowed researchers to make remarkable progress in the development of diagnostic tests, treatments and vaccines.

And the third lesson concerns government advice to schools, where the message is simply ‘Must do better’. As well as (the outstanding) work on biomedical science, there should have been more studies on mechanisms of transmission, on the role of masks, on ventilation and on social distancing, all of which would have allowed better decisions to have been made and better and (of great importance) more equitable outcomes for children.

## Data accessibility

This article has no additional data.
